# Differences between WHO Growth Standards and China Growth Standards in Assessing the Nutritional Status of Children Aged 0–36 Months Old

**DOI:** 10.3390/ijerph17010251

**Published:** 2019-12-30

**Authors:** Qianling Tian, Xiao Gao, Tingting Sha, Qiong He, Gang Cheng, Xialing Wu, Fan Yang, Xihong Wu, Cai Tang, Qunhui Xie, Yan Yan

**Affiliations:** Department of Epidemiology and Health Statistics, Xiangya School of Public Health, Central South University, Changsha 410078, China; 15274979382@163.com (Q.T.); 166901003@csu.edu.cn (X.G.); tingtingSha@csu.edu.cn (T.S.); qqniuniu0525@163.com (Q.H.); gangcheng@csu.edu.cn (G.C.); wuxl9404@163.com (X.W.); 176912057@csu.edu.cn (F.Y.); wuxihong@csu.edu.cn (X.W.); 186911018@csu.edu.cn (C.T.); xiequnan@163.com (Q.X.)

**Keywords:** growth standards, children, nutritional status, difference

## Abstract

*Background*: At present, whether to use the World Health Organization’s (WHO) growth standards or native growth standards to assess the nutritional status in a given population is unclear. This study aimed to compare the differences between the WHO’s growth standards and China’s growth standards in assessing the nutritional status of children aged 0~36 months. *Methods*: We used z-scores to evaluate the nutritional status of children. The weight-for-age z-scores (WAZs), length/height-for-age z-scores (LAZ/HAZs), and weight-for-length/height z-scores (WLZ/WHZs) were calculated using the WHO’s growth standards and China’s growth standards. MeNemar’s test was used to compare the nutritional status of children. *Results*: The results in this study showed that there were differences between the WHO’s standards and China’s standards in assessing children’s nutritional status except for stunting and obesity. The prevalence of underweight assessed using China’s standards was higher than when using the WHO’s standards (except when 3 and 36 months old). The prevalence of wasting was significantly higher when assessed using China’s standards than when using the WHO’s standards from 12 to 36 months. The prevalence of overweight was higher when assessed using the WHO’s standards from 3 to 8 months. *Conclusions*: Both the WHO’s and China’s growth standards are useful measures in assessing children’s nutritional status but with key significant differences. Therefore, caution should be taken in selecting appropriate measures in a given population.

## 1. Introduction

Children’s nutritional status is a reflection of national economic development and social civilization but also a vital measurement index of quality of life and quality of maternal and child health care for people. According to a recent report by the World Health Organization (WHO) [1], in 2018, there were still 1.49 million and 4.9 million children under the age of five who experienced stunting and wasting, respectively.

Physical development is an important index to assess children’s nutritional status primarily including weight and length/height [2]. The commonly used evaluation indexes are weight-for-age, length/height-for-age, weight-for-length/height, BMI-for-age, and head circumference-for-age [3]. The z-score is recommended by the WHO and is one of the most widely used methods to evaluate the growth and development of children [4,5]. It is also one of the most important methods to evaluate the growth, development, and nutritional status of children in China. Using the z-score method, it is possible to obtain the prevalence of overweight, obesity, wasting, stunting, and underweight of different children, and it is beneficial to compare children’s growth and nutritional status.

The growth standards are a unified scale for evaluating the growth and development of an individual or group of children or adolescents [6]. Different evaluation criteria will produce different evaluation results. In recent years, growth standards developed from healthy breastfed children have been used as the standards to evaluate the nutrition and health status of children which is of great public health significance for the prevention of overweight and obesity in children [7]. At present, the international recommended standards are the child growth standards issued by the WHO in April 2006 [3]. The widely used growth standards in China are the standards issued by the Ministry of Health in 2009 [8].

The WHO standards mainly include four indicators: weight-for-age, length/height-for-age, weight-for-height/length, and body mass index. Weight-for-age is an indicator of children’s short-term and long-term nutritional status. Length/height-for-age is an index to judge children’s long-term malnutrition. Weight-for-height/length is an index of acute malnutrition. The WHO growth standards were derived from the Multicentre Growth Reference Study (MGRS) conducted between 1997 and 2003. It was based on data from six countries (i.e., Brazil, Ghana, India, Norway, Oman, and the United States) including a longitudinal follow-up study of 0–24 months and a cross-sectional survey of children aged 18–71 months. It brought together a unified child growth assessment tool [9]. In setting the 2006 edition of the child growth standards, the WHO stated that the most important determinant of child growth is not heredity and race but nutrition, feeding methods, health care, and environmental factors [10] which are the basis for the worldwide promotion of the WHO’s standards. At the planning stage of the MGRS, the WHO working group conducted a study comparing the growth of breastfed children in Australia, Chile, China, Guatemala, India, Nigeria, and other countries. The results showed that the breastfeeding patterns of breastfed children from equally wealthy families in different countries were very similar. However, there were two exceptions: compared with Australia (reference), Chinese children were 3% shorter when they were 12 months old, and Indian children were 15% lighter [11]. Therefore, the WHO working group recommended adding samples from East and South Asia when setting standards. The WHO invited China to join the study, but China declined to participate; thus, there is a lack of samples from East Asia in the 2006 version of the WHO’s standards [12].

Since 1975, a sample survey on the growth and development of children in nine cities is organized every 10 years in China. The survey includes nine cities (i.e., Beijing, Harbin, Xi’an, Shanghai, Nanjing, Kunming, Wuhan, Guangzhou, and Fuzhou) which are distributed in the east, south, west, north, and middle of China [13,14,15]. The physical development standards of children in China are established by taking the measured values of children of different ages in nine cities as parameters. The latest child growth standards were developed by the Capital Institute of Pediatrics using data from the 5th Physical Development Survey implemented in nine cities in 2015. However, the weight, length/height, and head circumference of children under 3 years of age in the urban areas of the nine cities in 2015 did not increase significantly compared with 2005 [16]. This study still chooses the growth standards published in September 2009 for analysis [17]. The 2009 growth standards for Chinese children are the reference standards for the growth and development of children under 7 years of age in China. The data are derived from the physical Development Survey of Children under 7 years of age in nine cities in China from 2005. The length/height, weight, and head circumference of 69,760 healthy children were collected using a cross-sectional survey [18,19]. Same as the WHO’s growth standards, China’s growth standards also have percentile and standard deviation unit standard curves of children’s growth. The 2009 growth standards of Chinese children reflects the overall growth and development level of children in China, and it provides a reference for the evaluation of children’s growth and development in China.

In this study, we selected children born in three communities in Kaifu District, Changsha City, Hunan Province, in 2015 as research subjects. The length/height and weight data of children aged 1, 3, 6, 8, 12, 18, 24, and 36 months were collected prospectively. We used the 2006 edition of the WHO’s growth standards and the 2009 edition of China’s growth standards to calculate the z-score of each child. The objectives were to understand the nutritional status of children and compare the differences among the two standards to judge the children’s nutritional status.

## 2. Materials and Methods

### 2.1. Study Design and Population

**Figure 1:**
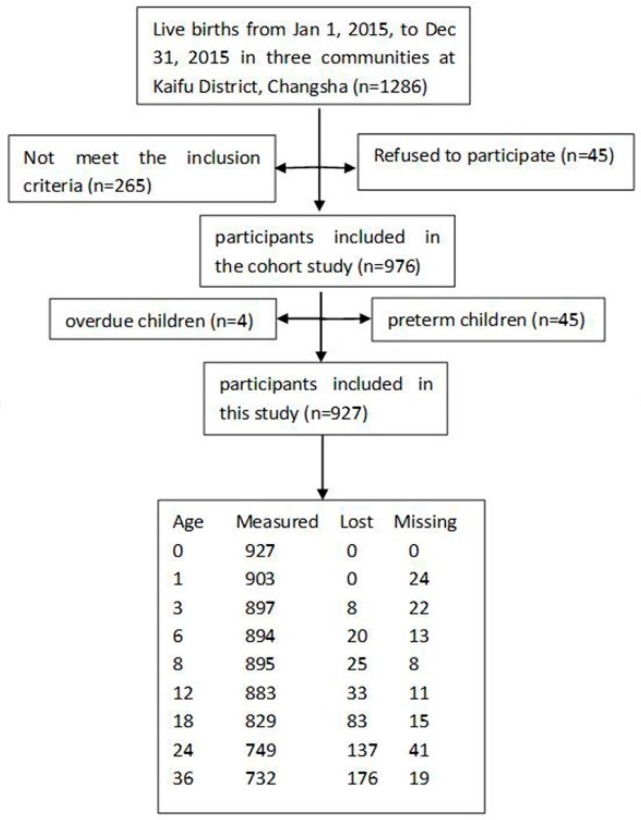
The participant flow diagram for the differences between the World Health Organization’s (WHO) growth standards and China’s growth standards in assessing the nutritional status of children aged 0–36 months old.

This study was from a birth cohort study conducted in Kaifu District, Changsha, China. The Community Health Service Centers of the Xinhelu, Dongfenglu, and Sifangping communities of the Kaifu District in Changsha, Hunan, China, were randomly selected as the investigation sites. A cluster sampling method was used to select the children who were born in these Community Health Service Centers during 2015. From 1 January 2015 to 31 December 2015, a total of 1286 infants were born in the three communities. The inclusion criteria of this prospective cohort study were as follows: (1) mothers and their children with completed records at any Community Health Service Centers; (2) participants who agreed to engage in our study and sign the informed consent form; (3) singleton births; (4) mothers who had no mental illnesses or brain diseases; and (5) children who had no congenital diseases. Finally, 976 eligible mother–child pairs were enrolled in the prospective cohort study. After excluding pre-term children (<37 weeks of gestation) and overdue children (>42 weeks of gestation), a total of 927 children were followed in the study between 2015 and 2018 in Changsha, China. Due to the fact that this was a cohort study, some of the children were lost to follow-up, and some of the children did not measure their weight and length/height at the Service Centers regularly, so the sample size was different for different months. The sample size at 0, 1, 3, 6, 8, 12, 18, 24, and 36 months old were 927, 903, 897, 894, 895, 883, 829, 750, and 732, respectively. The participant flow diagram of this study is shown in Figure 1.

### 2.2. Data Collection and Calculation

Service centers measured the weight and length/height of the children regularly from 0 to 36 months of age. The length was measured without shoes using the infantometer for children younger than 36 months old. Height was measured using a stadiometer for children aged 36 months (to the nearest 0.1 cm). Weight was measured without shoes and in light clothing using an electronic scale (to the nearest 0.1 kg). All equipment used in three communities were the same, and both length (or height) and weight were measured at eight target points twice to increase the reliability. We collected children’s weight and length/height from health care records in the Community Health Management Information System and child healthcare handbooks. 

The z-scores were calculated using the following formula [20]:

(1) $$Z-\mathrm{score}=\frac{{\left(X/M\right)}^{L}-1}{\mathrm{LS}}$$

where X is the physical measurement, and L, M, and S, respectively, denote the parameters lambda (the power needed to remove skewness, transforming the data into a normal distribution), mu, the population mean estimated by the observed 50th centile value for the measurement, and sigma, the coefficient of variation [21]. The application of this formula thus corrects for skewness in the reference data when calculating a z-score for a given sex, age, or length. According to the WHO’s standards, WAZ was between −6 and +5, LAZ/HAZ was between −6 and +6, and WHZ/WLZ was between −5 and +5. According to China’s growth standards, all the z-score values were between −5 and +5. 

### 2.3. Definitions

Stunting was defined as a LAZ/HAZ of <−2, and underweight was defined as a WAZ of <−2. Wasting was defined as a weight less than the corresponding weight of a WLZ of −2 for a particular length and sex. Overweight meant a weight more than the corresponding weight of a WLZ/WHZ of 2 for a specific length/height and sex. Obesity was defined as a weight heavier than the corresponding weight of a WLZ/WHZ of 3 for a particular length/height and sex [22].

### 2.4. Statistical Analysis

The data were checked manually for completeness and input via EpiData version 3.1 (EpiData Association, Odense, Denmark) by two investigators. All statistical analyses were performed using SPSS version 20 (IBM, New York, NY, USA). Continuous variables were described using mean ± standard deviation (SD), while categorical variables were described using percentage. A paired *t*-test was used to compare the WAZ, LAZ/HAZ, and WLZ/WHZ, and MeNemar’s test was used to compare the nutritional status in two different growth standards. *p*-Values < 0.05 were considered significant.

### 2.5. Ethical Considerations

The birth cohort study was approved by the Independent Ethics Committee Institute of Clinical Pharmacology, Central South University, Changsha, China.

## 3. Results

### 3.1. Weight and Length/Height

The weight and length/height of our study, China’s growth standards, and the WHO’s growth standards are presented in Table 1.

Compared to China’s growth standards, the difference in weight had statistical significance in boys 0 to 36 months old (except when 6 months old and 12 to 24 months old) (*p* < 0.05) and in girls 0 to 36 months old (except when 18 and 24 months old) (*p* < 0.05). Compared to the WHO’s growth standards, the weight in our study was relatively higher (*p* < 0.05) (Table 1).

Compared to China’s growth standards, for boys, the length/height in this study was higher at 1, 3, 8, and 36 months old (*p* < 0.05) but was lower at 0 and 12 months old (*p* < 0.05). For girls, the length/height in this study was higher at 1 to 3, 8, and 36 months old (*p* < 0.05); in other months, there was no statistical significance. Compared with the WHO’s growth standards, from birth to 36 months, the length/height of children in this study was higher (*p* < 0.05) (Table 1).

### 3.2. Z-Scores

The comparison of z-scores estimated by using China’s growth standards and the WHO’s growth standards in different months are shown in Table 2.

The WAZ assessed using the two standards in different months had a statistical difference. For boys, the WAZ assessed using the WHO’s standards was higher than when using China’s standards (except when 0 months old) (*p* < 0.05). For girls, the WAZ assessed using the WHO’s standards was higher than when using China’s standards as well (except when 0 and 1 months old) (*p* < 0.05) (Table 2).

The LAZ/HAZ for both boys and girls assessed using the WHO’s standards was higher than when using China’s standards. However, at 8 months old, the assessment using China’s standards was higher (*p* < 0.05) (Table 2).

The WLZs of children in this study were higher when assessed using the WHO’s standards from 3 to 36 months old. For 0 and 1 months old, the WLZs assessed using China’s standards were higher (*p* < 0.05) (Table 2).

### 3.3. Nutrition Status

Table 3 showed the nutrition status of children estimated using China’s standards and the WHO’s standards. The prevalence of underweight estimated using China’s growth standards was higher than when using the WHO’s growth standards from 0 to 24 months (except when 3 months old) (*p* < 0.05); at 3 and 36 months, there was no statistical difference. The prevalence of wasting assessed using China’s standards was higher than when using the WHO’s standards at 0 and 12 to 36 months as well (*p* < 0.05). The prevalence of stunting assessed using the two growth standards had no significant difference. The prevalence of overweight estimated using the WHO’s standards was higher than when using China’s standards from 3 to 8 months (*p* < 0.05); in other months, there was no significant difference. The prevalence of obesity estimated using the WHO’s standards and China’s standards had no significant difference, but at 0 and 36 months, assessed using China’s standards, it was higher than when using the WHO’s standards; in other months, assessed using the WHO’s standards, it was higher than when using China’s standards (Table 3).

**Figure 2:**
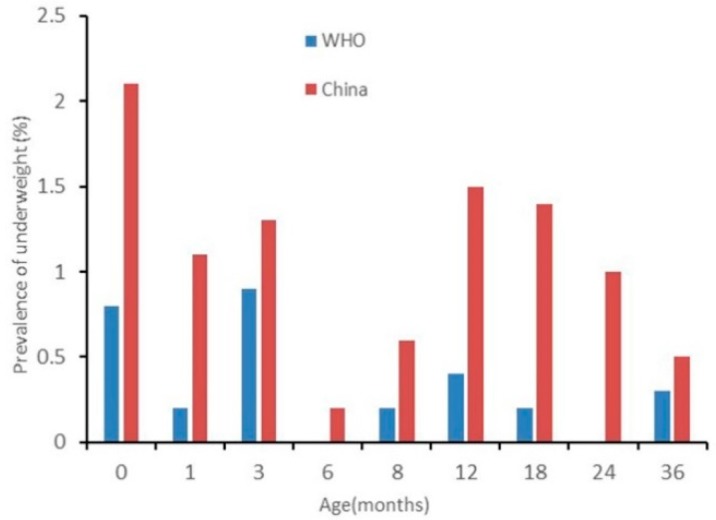
The difference in the prevalence of underweight for boys under 36 months assessed using the WHO’s growth standards and China’s growth standards.

**Figure 3:**
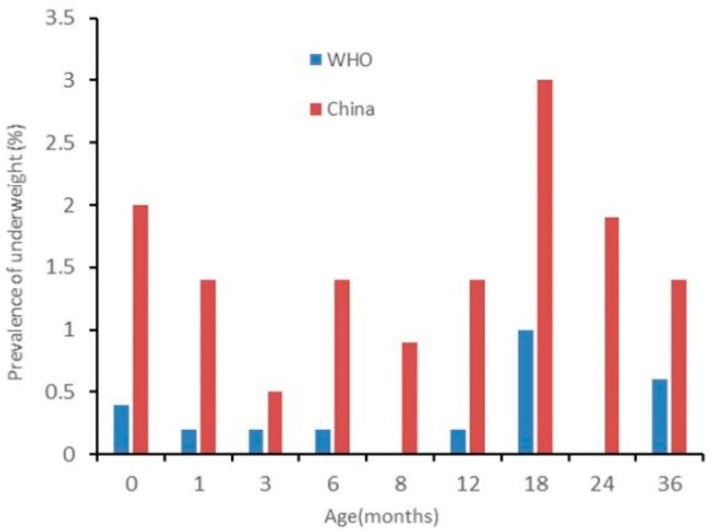
The difference in the prevalence of underweight for girls under 36 months assessed using the WHO’s growth standards and China’s growth standards.

**Figure 4:**
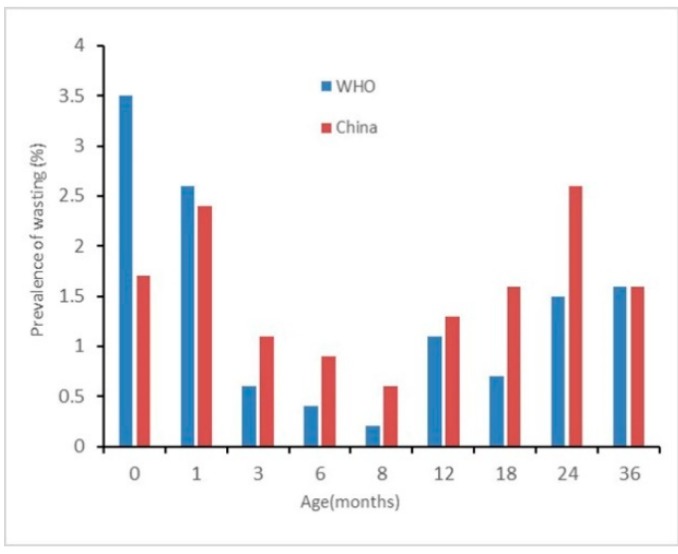
The difference in the prevalence of wasting for boys under 36 months assessed using the WHO’s growth standards and China’s growth standards.

**Figure 5:**
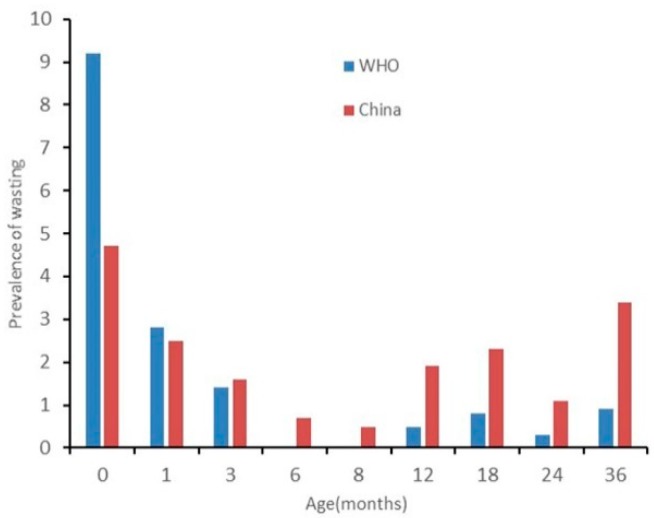
The difference in the prevalence of wasting for girls under 36 months assessed using the WHO’s growth standards and China’s growth standards.

**Figure 6:**
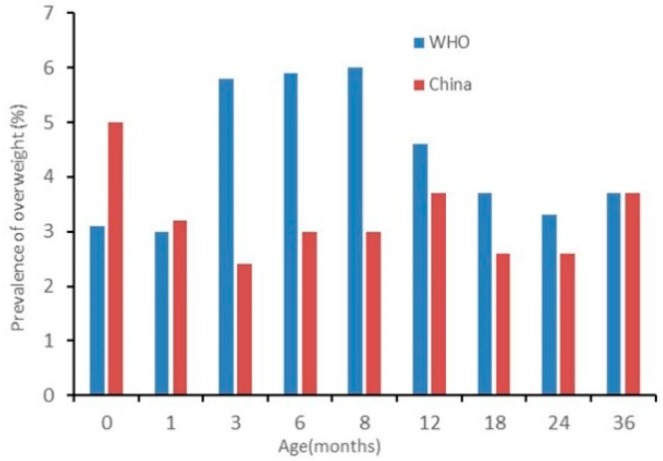
The difference in the prevalence of overweight for boys under 36 months assessed using the WHO’s growth standards and China’s growth standards.

**Figure 7:**
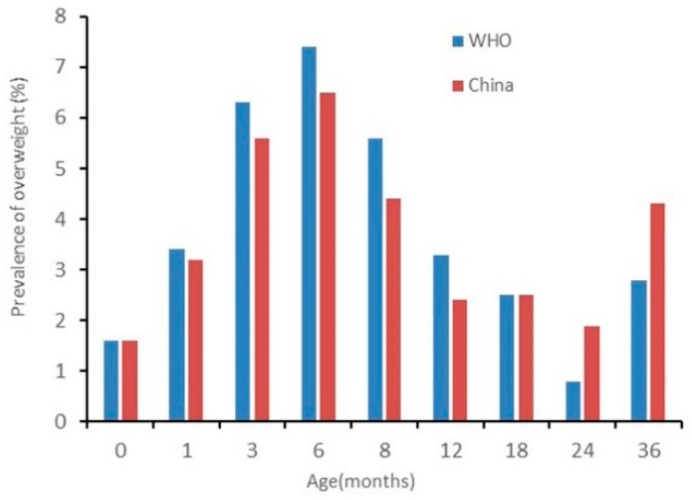
The difference in the prevalence of overweight for girls under 36 months assessed using the WHO’s growth standards and China growth’s standards.

The results of nutritional status by sex are presented in Figure 2, Figure 3, Figure 4, Figure 5, Figure 6 and Figure 7. The prevalence of stunting and obesity assessed using the two growth standards had no statistical significance, so we did not present the results concerning sex.

The prevalence of underweight for boys assessed using China’s standards was higher than when using the WHO’s standards from 0 to 36 months. However, it only had a significant difference when 0 months old (see Figure 2). For girls, the prevalence of underweight assessed using China’s standards was higher than when using the WHO’s standards, and it had a significant difference at 0, 18, and 24 months old (see Figure 3).

The prevalence of wasting for male children assessed using the WHO’s standards was higher than when using China’s standards at 0 months old (*p* < 0.05). For children 3 to 24 months old assessed using China’s standards, it was higher when using the WHO’s standards but had no statistical significance (see Figure 4). The prevalence of wasting for female children assessed using the WHO’s standards was higher than when using China’s standards for children 0 months old as well, but at 12, 18, and 36 months old, assessed using China’s standards, it was higher than when using the WHO’s standards (*p* < 0.05) (see Figure 5).

The prevalence of overweight for boys assessed using China’s standards was higher than when using the WHO’s standards at 0 months old. At 3 to 6 months old, assessed using the WHO’s standards, it was higher than when using China’s standards (*p* < 0.05) (see Figure 6). The prevalence of overweight in girls had no significant difference among the assessments when using the two standards (see Figure 7).

## 4. Discussion

The results in this study showed that there were some differences between the WHO’s standards and China’s standards in assessing children’s nutritional status except for stunting and obesity. The prevalence of underweight assessed using the two growth standards had a significant difference from 0 to 36 months (except when 3 and 36 months old). The prevalence of underweight was higher when assessed using China’s standards than when using the WHO’s standards. The prevalence of underweight for boys assessed using the two standards had no statistical significance; for girls assessed using China’s standards, it was higher than when using the WHO’s standards. The prevalence of wasting was significantly higher when assessed using China’s standards than when using the WHO’s standards from 12 to 36 months in girls. The prevalence of overweight was higher when assessed using the WHO’s standards from 3 to 8 months in boys. The number of girls who had weight-for-ages and weight-for-length/heights less than −2 standard deviation (SD) was lower when assessed using the WHO’s growth standards. However, the number of boys who had weight-for-length/heights more than +2SD was higher when assessed using the WHO’s growth standards. This means that the WHO’s growth standards can reduce the number of girls who have weight-for-ages and weight-for-length/heights less than −2SD and increase the number of boys who have weight-for-length/heights more than +2SD. If using the WHO’s growth standards to evaluate the prevalence of underweight and wasting, some girls and boys will not be adequately fed.

We used the WHO’s growth standards to assess the nutritional status of children in this study, and the prevalence of underweight, stunting, wasting, overweight, and obesity were 0.34%, 0.44%, 1.60%, 4.11%, and 0.53%, respectively. In addition, using China’s growth standards to assess the nutritional status of children in this study, the prevalence of underweight, stunting, wasting, overweight, and obesity were 1.31%, 0.65%, 1.78%, 3.40%, and 0.49%, respectively.

According to the results in this study, the weight and length/height were closer to China’s growth standards. The use of China’s growth standards to evaluate the growth of children in China may be more suitable. Some studies found that the WHO’s growth standards were not suitable for any region or age [23,24].

Many studies found that there were differences between the WHO’s standards and national standards in assessing children’s nutritional status. A global survey of the use of the new WHO standards found that of the 180 countries that responded, 125 adopted the new standards, 25 were under consideration, and 30 did not [25]. In addition to African countries, other countries are more inclined to use reference standards based on their populations [26,27]. On the other hand, studies have shown that there are differences between the new WHO standards and national standards in judging the growth and development of children [25]. Tanaka et al. compared the growth indicators of children aged 0–24 months in Japan with the national reference standards and the WHO’s growth standards [28]. They found that the length and weight of children aged 0–24 months were lower than the national reference standards and the WHO’s standards. However, on the whole, they were closer to the national reference standards. The results found by Tanaka et al. were not entirely consistent with the results of this study. The weights and length/heights in this study were higher than the WHO’s standards and were higher than the national standards in most months. Inokuchi et al. [29] found that the use of the WHO’s growth standards could increase the prevalence of stunting and overweight children aged 0–5 in Japan. In this study, we also found that when using the WHO’s standards and China’s standards, the overweight assessed using the WHO’s standards was higher than when using China’s standards. Juliusson et al. [30] combined the findings from Belgium and Norway and found that assessment using the WHO’s standards reduced the detection rate of underweight, wasting, and stunting, and increased the detection rate of overweight and obesity. In this study, we also found that the WHO’s standards can reduce the detection rate of underweight and wasting and increase the detection rate of overweight. The prevalence of stunting and obesity in our study was low. We did not find that the WHO’s standards were able to reduce the detection rate of stunting and increase the detection rate of obesity significantly. Yang et al. [31] also found that the prevalence of obesity assessed using the WHO’s and China’s standards had no significant difference. The Czech Republic compares the growth indicators of breastfed children with the national and WHO standard. It found that Czech children’s length/height-for-age percentage curve was generally higher than the WHO’s standards, and the weight was lower than the WHO’s standards in the previous months. However, for children over the age of 12 months, the weight-for-age was higher than the WHO’s standards [32].

Most studies have found that the weight and length/height in the national standard were higher than the WHO’s standards. British research on whether using the WHO’s standards shows that the WHO’s standards represents the growth standards of children under specific conditions, rather than reflecting the current growth level. Whether the WHO’s standards are adopted depends on the purpose of the study [33]. The WHO’s growth standards involve the growing trends of healthy children under the best environmental, medical, and hygienic conditions, and it provides an ideal target growth model [34]. The differences between the WHO’s growth standards and national growth standards were much the same in many countries which supports the statement that heredity and race are not the determinant factors. The study populations of the MGRS were in socioeconomic conditions that were favorable to growth and where mobility was low, and data on more than 20% of mothers who followed the WHO’s feeding recommendations and breastfeeding were available. The low birth weight (<2500 g) infants were not excluded from the WHO’s standards [9]. The study populations using China’s growth standards were from the survey on the growth and development of children in nine cities; not all the children were breastfed. The pre-term or low birth weight infants were excluded from China’s standards. The differences in inclusion and exclusion criteria of study populations may have caused the differences among the two growth standards.

The Centers for Disease Control and Prevention (CDC) growth charts have also been widely used. The weight-for-age in the CDC growth chart was higher than for the WHO’s standards which implies that the prevalence of underweight would be higher when using the CDC growth chart [35]. The CDC growth chart also has percentiles and z-scores curves [36], and the growth curves are based on children living in the United States. In some studies, they assessed the nutritional status of children based on the WHO’s growth standards. They found that the prevalence of obesity and overweight was very higher, so they suggested the corresponding department should take steps to reduce the prevalence of obesity. The physical development of children is affected by the level of local nutrition, feeding methods, health care, and environmental factors. The WHO’s growth standards are widely used around the world, and it can be used all over the world. When comparing differences in physical development and nutritional status among children in different countries, the WHO’s growth standards are a valuable tool. When assessing the nutritional status of local children and providing guidance, national references may be more fitting.

The limitation of this study was that the sample size was not very large; because of this, the numbers of stunting and obesity were low. The prevalence of stunting assessed using the WHO’s standards and China’s standards in other studies had a significant difference [31]. Whether the prevalence of stunting and obesity has a significant difference when assessed using the WHO’s growth standards and China’s standard should be researched with a larger sample size.

## 5. Conclusions

In conclusion, the weights and height/lengths in this study were higher than the WHO’s growth standards and China’s growth standards. The WAZ, HAZ/LAZ, and WLZ in this study were higher when assessed using the WHO’s growth standards than when using China’s growth standards. The prevalence of underweight and wasting assessed when using the WHO’s growth standards was higher than when using China’s growth standards, and the prevalence of overweight was higher when assessed using the WHO’s standards.

Both the WHO’s and China’s growth standards were useful measures in assessing children’s nutritional status but with key significant differences. Therefore, caution should be taken in selecting appropriate measures in a given population. Misjudgment of children’s nutritional status may lead to the wrong nutrition recommendations from the relevant departments which may affect children’s growth and development. The WHO’s growth standards can be used to evaluate breastfed children’s growth and development status and to compare the growth and development of children in different countries. When assessing the nutritional status of children, national growth standards may be more appropriate.

## Figures and Tables

**Table 1:** The weight and length/height of children from our study, China’s growth standards, and the WHO’s growth standards.

Age (month)	Weight (kg)						Length/Height (cm)					
	Boy			Girl			Boy			Girl		
	1	2	3	1	2	3	1	2	3	1	2	3
0	3.42	3.32 *	3.3 *	3.31	3.21 *	3.2 *	50.0	50.4 *	49.9 *	50.0	49.7 *	49.1 *
1	4.66	4.51 *	4.5 *	4.44	4.20 *	4.2 *	55.2	54.8 *	54.7 *	54.3	53.7 *	53.7 *
3	6.86	6.70 *	6.4 *	6.45	6.13 *	5.8 *	62.4	62.0 *	61.4 *	61.2	60.6 *	59.8 *
6	8.40	8.41	7.9 *	7.94	7.77 *	7.3 *	68.4	68.4	67.6 *	66.9	66.8	65.7 *
8	9.18	9.05 *	8.6 *	8.63	8.41 *	7.9 *	71.5	71.2 *	70.6 *	70.1	69.6 *	68.7 *
12	10.03	10.05	9.6 *	9.53	9.40 *	8.9 *	76.1	76.5 *	75.7 *	74.9	75.0	74.0 *
18	11.30	11.29	10.9 *	10.73	10.65	10.2 *	82.6	82.7	82.3 *	81.4	81.5	80.7 *
24	12.53	12.54	12.2 *	11.98	11.92	11.5 *	88.4	88.5	87.8 *	87.2	87.2	86.4 *
36	14.97	14.65 *	14.3 *	14.40	14.13 *	13.9 *	97.4	96.8 *	96.1 *	96.2	95.6 *	95.1 *

Note that 1 represents this study, 2 represents China’s growth standards, and 3 represents the World Health Organization’s (WHO) growth standards. The reference group was this study. * *p* < 0.05.

**Table 2:** Comparison of weight-for-age (WAZ), length/height-for-age (LAZ/HAZ), and weight-for-length/height (WLZ/WHZ) estimated using China’s growth standards and the WHO’s growth standards in different months. Data from the Kaifu District, Changsha, Hunan, China, 2015.

Age (month)	WAZ (Mean ± SD)						LAZ/HAZ (Mean ± SD)						WLZ (Mean ± SD)					
	Boy			Girl			Boy			Girl			Boy			Girl		
	WHO	China	*p*	WHO	China	*p*	WHO	China	*p*	WHO	China	*p*	WHO	China	*p*	WHO	China	*p*
0	0.12 ± 0.80	0.22 ± 0.98	0.000	0.15 ± 0.88	0.19 ± 1.03	0.000	0.06 ± 0.36	−0.22 ± 0.39	0.000	0.44 ± 0.32	0.14 ± 0.35	0.000	0.21 ± 1.17	0.49 ± 1.10	0.000	−0.21 ± 1.26	0.02 ± 1.18	0.000
1	0.28 ± 0.80	0.25 ± 0.90	0.000	0.39 ± 0.84	0.40 ± 0.96	0.077	0.22 ± 0.90	0.17 ± 0.84	0.000	0.34 ± 0.86	0.32 ± 0.84	0.000	0.23 ± 1.05	0.25 ± 1.00	0.000	0.21 ± 1.05	0.26 ± 1.03	0.000
3	0.59 ± 0.88	0.17 ± 0.89	0.000	0.71 ± 0.86	0.38 ± 0.93	0.000	0.47 ± 1.02	0.17 ± 0.91	0.000	0.66 ± 0.98	0.26 ± 0.92	0.000	0.50 ± 1.02	0.24 ± 0.96	0.000	0.48 ± 1.03	0.38 ± 1.04	0.000
6	0.48 ± 0.90	−0.05 ± 0.87	0.000	0.62 ± 0.88	0.13 ± 0.97	0.000	0.35 ± 1.01	−0.02 ± 0.90	0.000	0.52 ± 0.94	0.05 ± 0.93	0.000	0.53 ± 0.98	0.15 ± 0.93	0.000	0.63 ± 0.90	0.36 ± 0.98	0.000
8	0.55 ± 0.92	0.09 ± 0.89	0.000	0.62 ± 0.88	0.17 ± 0.98	0.000	0.40 ± 0.99	0.65 ± 0.88	0.000	0.56 ± 0.97	0.76 ± 0.95	0.000	0.58 ± 0.95	0.23 ± 0.92	0.000	0.59 ± 0.90	0.30 ± 0.99	0.000
12	0.31 ± 0.91	−0.07 ± 0.89	0.000	0.46 ± 0.84	0.07 ± 0.96	0.000	0.14 ± 0.99	−0.18 ± 0.86	0.000	0.34 ± 0.94	−0.04 ± 0.89	0.000	0.37 ± 0.95	0.09 ± 0.95	0.000	0.46 ± 0.91	0.18 ±1.02	0.000
18	0.25 ± 0.92	−0.04 ± 0.93	0.000	0.33 ± 0.85	0.01 ± 0.98	0.000	0.11 ± 1.04	−0.04 ± 0.90	0.000	0.24 ± 0.96	−0.05 ± 0.92	0.000	0.31 ± 0.97	0.03 ± 1.00	0.000	0.31 ± 0.93	0.08 ± 1.06	0.000
24	0.23 ± 0.86	−0.06 ± 0.90	0.000	0.29 ± 0.83	−0.02 ± 0.95	0.000	0.17 ± 0.95	−0.06 ± 0.82	0.000	0.25 ± 0.97	−0.02 ± 0.90	0.000	0.19 ± 0.94	0.02 ± 0.98	0.000	0.17 ± 0.83	0.08 ± 0.96	0.000
36	0.30 ± 0.91	0.13 ± 0.99	0.000	0.23 ± 0.89	0.09 ± 1.03	0.000	0.35 ± 0.95	0.14 ± 0.91	0.000	0.30 ± 0.93	0.14 ± 0.93	0.000	0.19 ± 1.04	0.10 ± 1.11	0.000	0.09 ± 1.00	0.05 ± 1.14	0.000

Note: WHO represents the WHO’s growth standards, and China represents China’s growth standards.

**Table 3:** The rates of nutritional status assessed using the WHO’s growth standards and China’s growth standards.

Age (months)	Underweight *n* (%)			Stunting *n* (%)			Wasting *n* (%)			Overweight *n* (%)			Obesity *n* (%)		
	WHO	China	*p*	WHO	China	*p*	WHO	China	*p*	WHO	China	*p*	WHO	China	*p*
0	6 (0.6)	19 (2.0)	0.000	2 (0.2)	2 (0.2)	1.000	58 (6.3)	29 (3.1)	0.000	22 (2.4)	31 (3.3)	0.064	6 (0.6)	11 (1.2)	0.063
1	2 (0.2)	11 (1.2)	0.004	4 (0.4)	4 (0.4)	1.000	24 (2.7)	22 (2.4)	0.500	29 (3.2)	29 (3.2)	1.000	5 (0.6)	6 (0.7)	1.000
3	5 (0.6)	8 (0.9)	0.250	6 (0.7)	9 (1.0)	0.250	9 (1.0)	12 (1.3)	0.508	54 (6.0)	33 (3.9)	0.000	6 (0.7)	5 (0.6)	1.000
6	1 (0.1)	7 (0.8)	0.031	5 (0.6)	8 (0.9)	0.250	2 (0.2)	7 (0.8)	0.063	59 (6.6)	42 (4.7)	0.000	4 (0.4)	1 (0.1)	0.250
8	1 (0.1)	7 (0.8)	0.031	2 (0.2)	2 (0.2)	1.000	1 (0.1)	5 (0.6)	0.125	52 (5.8)	33 (3.7)	0.000	7 (0.8)	4 (0.4)	0.250
12	3 (0.3)	13 (1.5)	0.002	2 (0.2)	6 (0.7)	0.125	7 (0.8)	14 (1.6)	0.016	35 (4.0)	27 (3.1)	0.057	6 (0.7)	3 (0.3)	0.250
18	5 (0.6)	18 (2.2)	0.000	7 (0.8)	8 (1.0)	1.000	6 (0.7)	16 (1.9)	0.002	26 (3.1)	21 (2.5)	0.125	3 (0.4)	2 (0.2)	1.000
24	0 (0.0)	11 (1.5)	0.001	4 (0.5)	6 (0.8)	0.500	7 (0.9)	14 (1.9)	0.016	16 (2.1)	17 (2.3)	1.000	1 (0.1)	1 (0.1)	1.000
36	3 (0.4)	7 (1.0)	0.125	2 (0.3)	5 (0.7)	0.250	9 (1.2)	18 (2.5)	0.004	24 (3.3)	29 (4.0)	0.180	3 (0.4)	5 (0.7)	0.500

Note: WHO represents the WHO’s growth standards, and China represents China’s growth standards.
